# Cordycepin Ameliorates Constant Light-Induced Thermogenic Dysfunction in Brown Adipose Tissue by Activating SIRT1-Mediated Mitochondrial Homeostasis

**DOI:** 10.3390/ijms27104351

**Published:** 2026-05-13

**Authors:** Yonghui Bi, Guanyu Zhang, Yibing Wang, Li Zhang, Shuai Wu, Yongqiang Zhang, Xi Li, Danfeng Yang

**Affiliations:** 1 Academy of Military Medical Sciences, Academy of Military Sciences, Tianjin 300050, China; 15039463903@163.com (Y.B.); zhangguanyu555@foxmail.com (G.Z.); 13007036760@163.com (Y.W.); lizhang850115@163.com (L.Z.); wushuai_amms@163.com (S.W.); freechinese1984@163.com (Y.Z.); 2 Graduate School, Tianjin University of Traditional Chinese Medicine, Tianjin 301617, China

**Keywords:** cordycepin, constant light exposure, SIRT1 activation, brown adipose tissue whitening

## Abstract

Constant light (LL) exposure is an established environmental risk factor for metabolic diseases, in which the whitening of brown adipose tissue (BAT) plays a critical role. This study aimed to elucidate the molecular mechanisms through which cordycepin counteracts LL-induced BAT whitening and improves metabolic function. We established an LL-exposed mouse model and employed an integrative approach combining pharmacological, metabolic, molecular, and computational (docking) assays to define cordycepin’s effects and targets. Cordycepin treatment significantly improved cold tolerance and attenuated BAT whitening in LL mice. Mechanistically, cordycepin directly bound to and enhanced the activity of the NAD^+^-dependent deacetylase SIRT1. This activation mitigated LL-induced impairments in mitochondrial biogenesis, dynamics, and autophagy. Furthermore, SIRT1 activation rebalanced fatty acid metabolism by downregulating CD36 and upregulating CPT1, thereby restoring the coupling of fatty acid uptake to oxidation. All beneficial effects of cordycepin were abolished by the selective SIRT1 inhibitor EX-527. In summary, our work provides strong evidence that cordycepin directly interacts with SIRT1 and enhances its deacetylase activity, thereby restoring mitochondrial function and fatty acid oxidative homeostasis in BAT to counteract constant LL-induced metabolic dysfunction. These findings position cordycepin as a promising natural compound targeting the SIRT1 pathway for metabolic disorders.

## 1. Introduction

The spread of artificial light at night (ALAN) and associated shift-work patterns has raised significant public health concerns due to the established link of these factors to metabolic disorders, a connection primarily mediated through circadian rhythm disruption [1,2]. Constant light (LL) is recognized as a potent environmental circadian disruptor, which has been experimentally and epidemiologically linked to an elevated risk of metabolic disorders, including obesity and dyslipidemia [3,4,5,6]. In this pathological context, the functional status of brown adipose tissue (BAT) has garnered significant research interest [7]. Although once thought to be relevant primarily to infants and small mammals, thermogenically active BAT is now recognized as a functional and metabolically significant organ in healthy adults. Its major depots are located in the cervical and supraclavicular regions [8,9]. BAT activity correlates positively with whole-body energy expenditure and inversely with cardiometabolic risk markers, such as body mass index (BMI) and fasting glucose. This relationship underscores its role in maintaining systemic metabolic homeostasis [10,11,12]. Consequently, the preservation of BAT function is crucial for metabolic health. Pathologically, BAT undergoes “whitening”, a process characterized by ectopic lipid accumulation, mitochondrial depletion, and loss of thermogenic capacity. This pathological transformation is established as a key event in metabolic disease progression [13,14,15]. Notably, environmental circadian disruptors, in particular constant light (LL) exposure, have been identified as significant inducers of BAT whitening and thermogenic dysfunction [16]. Despite this understanding, the upstream molecular drivers of LL-induced BAT whitening, particularly the core and druggable targets within this network, remain poorly characterized.

The efficiency of BAT thermogenesis is reliant upon the coordinated rapid uptake and oxidation of circulating fatty acids [17,18,19]. This metabolic program is coordinated by the SIRT1–PGC-1α signaling axis [20,21]. SIRT1, a core NAD^+^-dependent deacetylase and cellular energy sensor, activates the transcriptional coactivator PGC-1α. Upon activation, PGC-1α drives the expression of genes critical for mitochondrial biogenesis and fatty acid oxidation, including CPT1β [21,22,23]. The integrity of this SIRT1–PGC-1α axis is therefore essential for aligning mitochondrial oxidative capacity to substrate supply [21,24,25]. AMP-activated protein kinase (AMPK) is another master sensor of cellular energy status that directly regulates fatty acid oxidation through phosphorylation of acetyl-CoA carboxylase (ACC), thereby modulating malonyl-CoA levels and CPT1 activity [26,27]. Importantly, AMPK and SIRT1 engage in extensive crosstalk: AMPK activation enhances SIRT1 activity by increasing NAD^+^ availability, while SIRT1 can deacetylate and activate LKB1, the upstream kinase of AMPK [28,29]. This positive feedback loop coordinates cellular energy metabolism and mitochondrial function. When this integrated energy-sensing network is impaired, and CD36-mediated fatty acid uptake persists without compensatory downregulation, it creates a critical mismatch between oxidative capacity and substrate influx. This imbalance precipitates lipotoxicity, thermogenic failure, and ultimately, tissue whitening. Given that SIRT1 activity is under robust circadian control [30], it was hypothesized that LL, as a circadian disruptor, induces BAT dysfunction primarily by suppressing the SIRT1–PGC-1α axis, thereby initiating this pathological cascade.

Natural product interventions, aligning with the “food as medicine” paradigm, offer a promising multi-target and low-toxicity strategy for metabolic diseases [31,32]. Cordycepin (3’-deoxyadenosine), a bioactive nucleoside derived from *Cordyceps militaris*, exhibits diverse pharmacological properties [33,34,35] and has been shown to mitigate LL-induced BAT whitening [36]. However, the precise molecular target(s) and downstream mechanisms through which cordycepin exerts this protective effect have remained largely unknown.

Therefore, this study was designed to elucidate the molecular mechanism by which cordycepin modulates SIRT1 signaling to reverse LL-induced BAT whitening. We hypothesized that cordycepin directly interacts with SIRT1, leading to enhanced deacetylase activity and subsequent restoration of mitochondrial function. By establishing that cordycepin directly interacts with SIRT1 to restore BAT function, our work provides a novel therapeutic target for light-associated metabolic disorders and offers new insights into their underlying pathology.

## 2. Results

### 2.1. Cordycepin Ameliorates Constant Light-Induced Metabolic Disturbances and Defective Brown Adipose Tissue Thermogenesis

**Figure 1:**
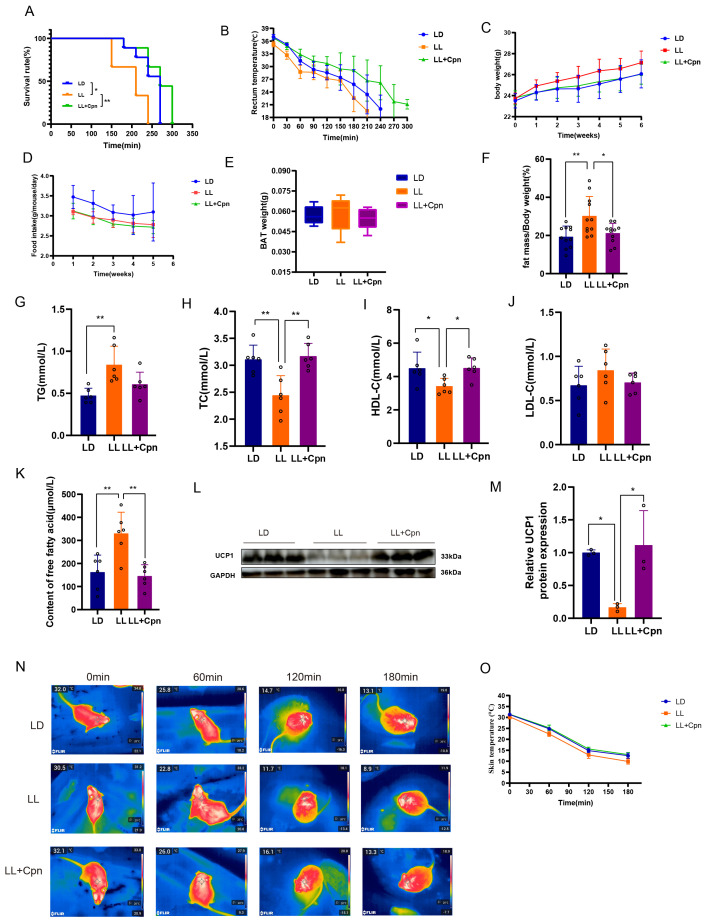
Cordycepin ameliorates systemic energy metabolism disorders induced by constant light exposure. Male C57BL/6J mice were subjected to a 12:12 h light–dark cycle (LD) or constant light (LL) for 5 weeks. A subset of LL mice was treated daily with cordycepin (Cpn, 50 mg/kg). (**A**) Kaplan–Meier survival curves during an acute cold challenge (−5 °C; *n* = 9 mice per group); (**B**) core body temperature (*n* = 9 mice per group); (**C**) weekly body weight changes (*n* = 11 mice per group); (**D**) average daily food intake; (**E**) BAT weight (*n* = 11 mice per group); (**F**) body fat percentage at the end of the 5-week experiment (*n* = 11 mice per group). (**G**–**K**) Serum levels of triglycerides (TG), total cholesterol (TC), high-density lipoprotein cholesterol (HDL-C), low-density lipoprotein cholesterol (LDL-C), and free fatty acids (FFA) (*n* = 6 mice per group). (**L**,**M**) Representative Western blot and densitometric quantification of UCP1 protein levels (*n* = 3 mice per group). (**N**) Dorsal skin temperature over the interscapular region was monitored using infrared thermography. (**O**) Quantitative analysis of interscapular skin temperature during cold exposure (*n* = 9 mice per group). Data are presented as mean ± SD with individual data points shown. Statistical significance was determined by log-rank (Mantel–Cox) test for survival curves and one-way ANOVA followed by Tukey’s post hoc test for all other comparisons (* *p* < 0.05, ** *p* < 0.01).

To systematically evaluate the impact of LL exposure on whole-body metabolism and the therapeutic effect of cordycepin (Cpn), we analyzed basic metabolic parameters. Phenotypically, although no significant differences in body weight or food intake were observed, LL exposure significantly increased the body fat percentage in mice, an effect that was reversed by Cpn treatment (Figure 1C–F). This suggests that LL induces lipid metabolic dysregulation. Further serum lipid profiling revealed a dyslipidemic profile in LL-exposed mice, characterized by elevated triglyceride (TG) levels and decreased levels of total cholesterol (TC) and high-density lipoprotein cholesterol (HDL-C). No significant change in low-density lipoprotein cholesterol (LDL-C) was observed. Cpn administration reversed the reductions in TC and HDL-C; however, it did not produce a statistically significant improvement in the elevated TG levels (Figure 1G–J). Moreover, serum levels of free fatty acids (FFA), the primary fuel for BAT thermogenesis [37], were markedly increased in the LL group and were restored by Cpn intervention (Figure 1K). This finding suggests that LL may specifically impair the body’s capacity for FFA clearance and utilization.

Given the close link between FFA clearance and thermogenesis, we assessed thermogenic function via an acute cold challenge to determine if it was impaired by LL. Our results showed that under cold stress, LL-exposed mice exhibited a rapid decline in core body temperature and a significantly reduced survival time, indicating a severely compromised thermogenic capacity. Cpn treatment significantly improved cold tolerance, better maintained core body temperature, and extended survival time (Figure 1A,B,N,O). This functional evidence confirms that Cpn can reverse the LL-induced thermogenic defect.

To directly assess BAT thermogenic capacity at the molecular level, we measured the protein expression of uncoupling protein 1 (UCP1), the key effector of BAT thermogenesis. As shown in (Figure 1L,M), UCP1 expression was markedly reduced in LL-exposed BAT compared to the LD control group. Notably, Cpn treatment restored UCP1 protein levels to those observed in the LD group. These results provide molecular evidence that Cpn rescues LL-induced thermogenic dysfunction by restoring UCP1 expression, consistent with the improved cold tolerance observed in Cpn-treated mice.

Taken together, these data demonstrate that chronic LL exposure induces systemic metabolic dysregulation, characterized by defective thermogenesis and disrupted FFA homeostasis, both of which were effectively ameliorated by Cpn.

### 2.2. Cordycepin Reverses BAT Whitening by Restoring Fatty Acid Uptake–Oxidation Coupling

**Figure 2:**
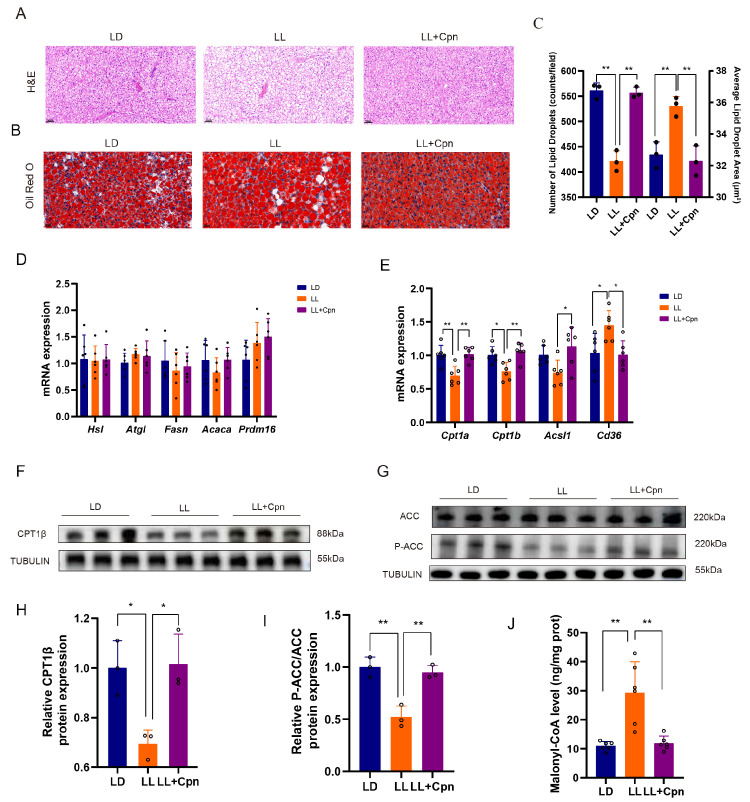
Cordycepin reverses BAT whitening by rebalancing the dysregulated fatty acid uptake and oxidation. (**A**) Representative hematoxylin and eosin (HE) staining images of BAT sections. Scale bar: 50 μm. (**B**) Representative images of Oil Red O staining for neutral lipid visualization. Scale bar: 20 μm. (**C**) Quantification of the average lipid droplet area from Oil Red O images; (**D**) relative mRNA expression of genes related to lipogenesis (Fasn, Acaca) and lipolysis (Atgl, Hsl, Prdm16); (**E**) relative mRNA expression of the fatty acid transporter Cd36 and mitochondrial β-oxidation enzymes Cpt1α, Cpt1β and Acsl1; (**F**,**H**) representative Western blot and densitometric quantification of CPT1β protein levels; (**G**,**I**) representative Western blot and quantification of the ratio of phosphorylated ACC (p-ACC) to total ACC (t-ACC). (**J**) Malonyl-CoA levels in BAT tissue measured. Tubulin was used as a loading control for Western blots. Data are presented as mean ± SD with individual data points shown (*n* = 3 mice per group for Western blot; *n* = 6 mice per group for qPCR; *n* = 6 mice per group for malonyl-CoA measurement). Statistical significance was determined by one-way ANOVA with Tukey’s post hoc test (* *p* < 0.05, ** *p* < 0.01).

To investigate the mechanism underlying impaired FFA clearance, we focused on brown adipose tissue (BAT) as a key metabolic site. Histological analysis confirmed that LL exposure induced profound BAT “whitening”, characterized by the accumulation of large, unilocular lipid droplets (Figure 2A–C). To trace the source of this ectopic lipid, the expression of key genes involved in de novo lipogenesis (Fasn, Acaca) and lipolysis (Atgl, Hsl, Prdm16) was assessed. Their mRNA levels remained unchanged across groups (Figure 2D), suggesting that the lipid accumulation in LL mice and its reversal by Cpn are not primarily mediated by transcriptional reprogramming of these canonical pathways.

Based on this finding, we shifted our focus to the balance between fatty acid uptake and oxidation. The analysis revealed a core dysregulation favoring lipid accumulation: at the transcriptional level, LL exposure significantly upregulated Cd36 (encoding a key fatty acid transporter) while downregulating Cpt1α and Cpt1β (genes for the rate-limiting enzyme of mitochondrial β-oxidation) (Figure 2E). This transcriptional suppression was confirmed at the protein level, where CPT1 β expression was markedly reduced in LL mice and robustly restored by Cpn intervention (Figure 2F,H).

We further examined the upstream enzymatic regulation. Acetyl-CoA carboxylase (ACC) catalyzes the production of malonyl-CoA, a potent allosteric inhibitor of CPT1 [38,39]. Phosphorylation of ACC (p-ACC) inhibits its activity, thereby relieving CPT1 inhibition [40,41]. The results indicated that LL significantly decreased the p-ACC/ACC ratio, suggesting enhanced ACC activity and consequently greater suppression of fatty acid oxidation. Cpn treatment fully restored this ratio (Figure 2G,I).

To directly assess the functional consequence of altered ACC phosphorylation, we measured malonyl-CoA levels in BAT tissue. As shown in (Figure 2J), malonyl-CoA levels were significantly elevated in the LL group compared to the LD control, consistent with the decreased p-ACC/ACC ratio. This elevation provides a direct mechanistic explanation for the reduced CPT1β expression and impaired fatty acid oxidation in LL-exposed BAT. Importantly, Cpn treatment restored malonyl-CoA levels to the LD control, indicating normalization of ACC activity and relief of CPT1 inhibition.

Based on these coordinated disruptions—increased uptake, decreased oxidation, and enhanced enzymatic inhibition—it is proposed that LL induces a state of impaired fatty acid flux in BAT. This state is characterized by a critical mismatch wherein fatty acid influx exceeds mitochondrial oxidative capacity, leading to lipid accumulation and tissue whitening. Cpn counteracts this pathology by restoring metabolic homeostasis.

### 2.3. Cordycepin Enhances the SIRT1–PGC-1α Axis via Direct and Indirect Mechanisms

**Figure 3:**
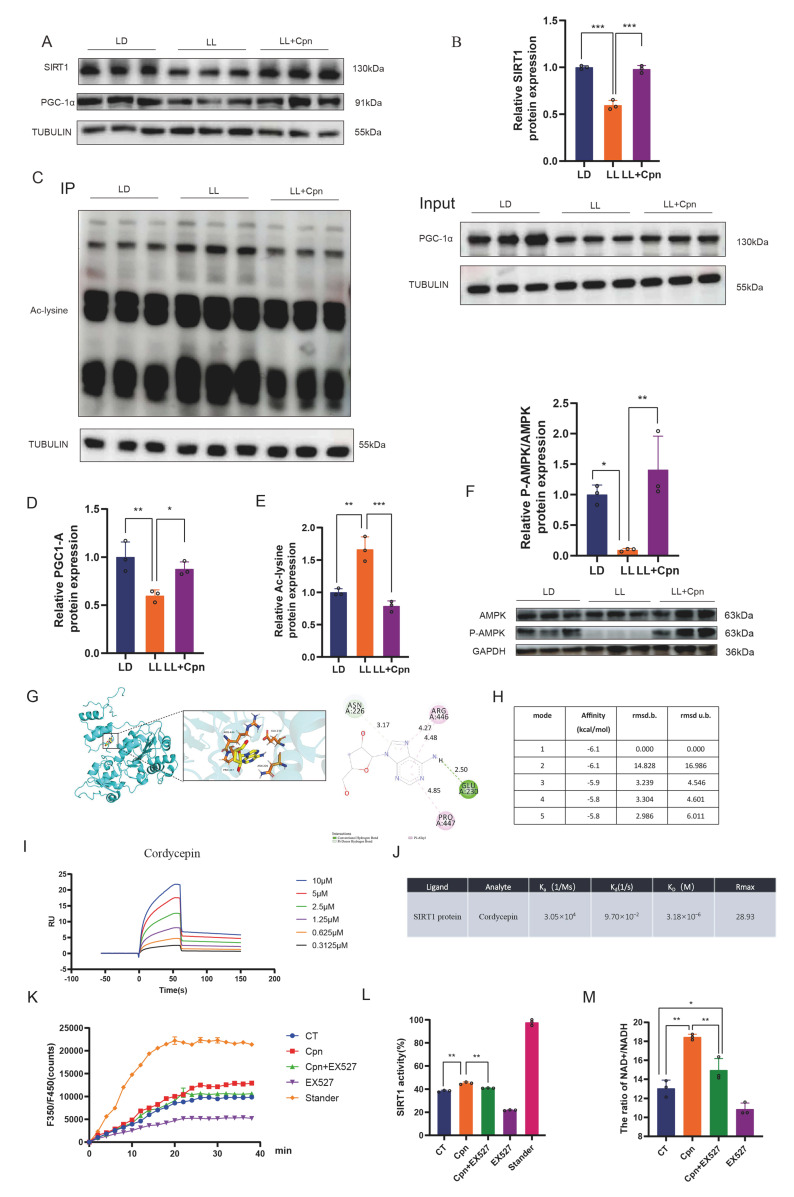
Cordycepin enhances the SIRT1-PGC-1α signaling axis via a dual mechanism involving direct binding and indirect modulation of NAD^+^. (**A**,**C**) Representative Western blots of SIRT1, acetylated PGC-1α (Ac-PGC-1α), and total PGC-1α in BAT lysates; (**B**,**D**–**E**) densitometric quantification of SIRT1,Ac-PGC-1α and total PGC-1α; (**F**) representative Western blot and densitometric quantification of P-AMPK/AMPK protein levels; (**G**,**H**) in silico molecular docking simulations illustrating the predicted binding pose of Cpn within the catalytic pocket of human SIRT1 with key interacting residues highlighted; (**I**,**J**) Surface Plasmon Resonance (SPR) analysis showing the direct physical interaction between Cpn and recombinant human SIRT1 protein with representative sensorgram (**I**) and steady-state affinity analysis (**J**) shown. The equilibrium dissociation constant (KD) was calculated; (**K**–**M**) intracellular NAD^+^/NADH ratio (**M**) and SIRT1 deacetylase activity (**K**,**L**), measured in primary brown adipocytes treated with Cpn (Cpn, 10 μM) and/or the SIRT1-specific inhibitor EX-527 (10 μM) for 48 h. Tubulin was used as a loading control for Western blots. Data are presented as mean ± SD with individual data points shown (*n* = 3 mice per group for Western blot; *n* = 3 independent experiments for in vitro studies). Statistical significance was determined by one-way ANOVA with Tukey’s post hoc test (* *p* < 0.05, ** *p* < 0.01, *** *p* < 0.001).

To explore the mechanism by which Cpn alleviates metabolic dysregulation, the SIRT1–PGC-1α axis, a master regulator of mitochondrial function and lipid oxidation [28], was investigated. Western blot analysis revealed that LL exposure significantly reduced total SIRT1 protein levels compared with the LD control. Concurrently, PGC-1α acetylation was markedly increased, indicating its functional inactivation. Cpn intervention not only restored SIRT1 expression but also significantly reduced PGC-1α acetylation (Figure 3A–E), thereby reactivating this key transcriptional coactivator.

Given that cordycepin is known to activate AMPK [42,43], we next examined AMPK phosphorylation at Thr172. As shown in (Figure 3F), chronic LL exposure significantly reduced the p-AMPK/AMPK ratio compared to the LD control group, indicating suppressed AMPK activity. Notably, Cpn treatment fully restored AMPK phosphorylation to levels comparable to the LD group. These results demonstrate that Cpn activates AMPK signaling in BAT of LL-exposed mice.

To assess the functional consequences of AMPK activation, we examined its downstream targets. AMPK directly phosphorylates ACC at Ser79, leading to its inhibition and subsequent reduction in malonyl-CoA levels, thereby relieving CPT1-mediated fatty acid oxidation [38,40]. Consistent with this, our data in (Figure 2F–J) show that Cpn restored ACC phosphorylation, normalized malonyl-CoA levels, and upregulated CPT1β expression in LL-exposed BAT.

Importantly, AMPK and SIRT1 form a positive feedback loop: AMPK activation increases NAD^+^ levels, enhancing SIRT1 activity [20,28]. The restoration of AMPK phosphorylation by Cpn therefore not only directly contributes to the observed improvements in fatty acid oxidation, but also synergizes with SIRT1 activation to amplify the metabolic benefits.

To determine how Cpn binds to SIRT1, we performed molecular docking simulations using the crystal structure of human SIRT1 (PDB ID: 5BTR). The simulation predicted that Cpn binds to SIRT1, forming conventional hydrogen bonds with key residues including ASN 226, ARG 446, GLU 230, and PRO 447 (Figure 3G,H). This interaction was confirmed using surface plasmon resonance (SPR), which demonstrated direct, concentration-dependent binding between Cpn and recombinant human SIRT1 (Figure 3I,J). Nevertheless, the predicted binding site(s) of Cpn on SIRT1 require further experimental validation.

Furthermore, since SIRT1 activity depends critically on intracellular NAD^+^ levels [20], we measured the NAD^+^/NADH ratio in primary brown adipocytes to assess the relevant cofactor milieu. Cpn treatment significantly increased this ratio and, concomitantly, enhanced SIRT1 deacetylase activity (Figure 3K–M). Together, these results demonstrate that Cpn enhances SIRT1 activity through a dual mechanism: direct physical interaction with the enzyme, coupled with an increase in cellular NAD^+^ bioavailability that potentiates its catalytic function. While these data support a functional interaction between Cpn and SIRT1, definitive proof of direct catalytic activation would require additional biochemical assays. The concurrent activation of AMPK by Cpn provides an additional upstream mechanism that synergistically enhances SIRT1 activity, forming a coordinated AMPK–SIRT1 signaling axis in response to metabolic stress.

### 2.4. Cordycepin Ameliorates Constant Light-Induced Impairment of Mitochondrial Quality Control

**Figure 4:**
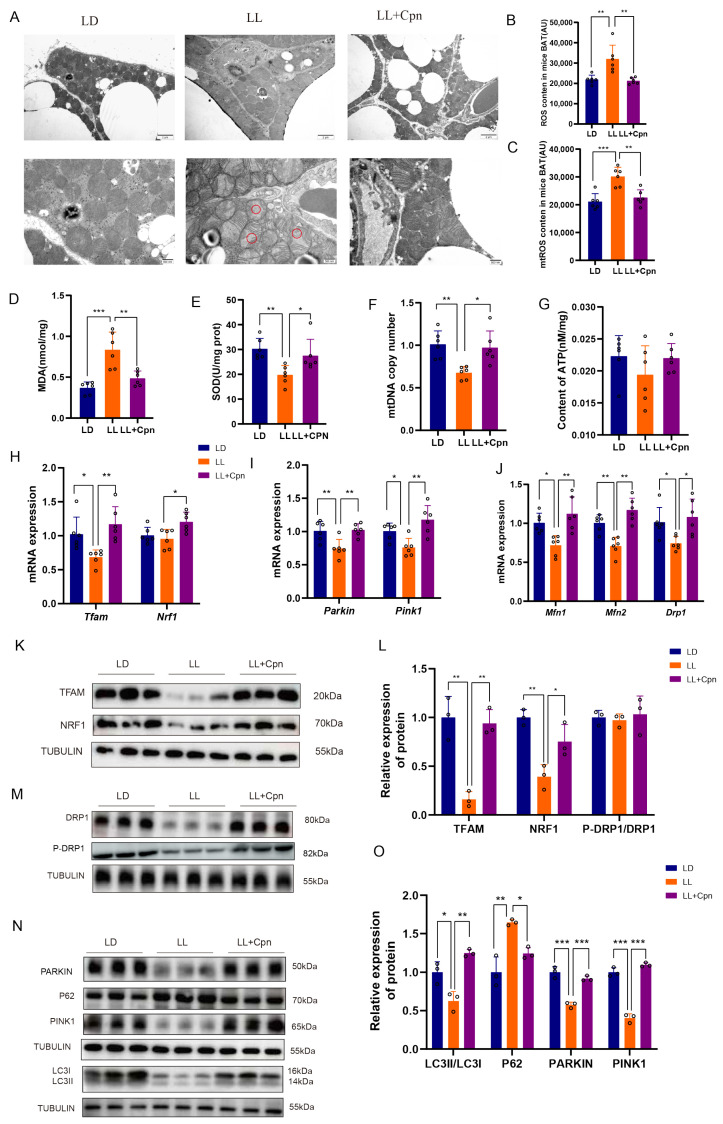
Cordycepin comprehensively restores mitochondrial quality control and homeostasis in the brown adipose tissue of mice exposed to constant light. (**A**) Representative transmission electron microscopy (TEM) images showing mitochondrial ultrastructure. Circles highlight damaged mitochondria exhibiting fragmented cristae and vacuolization. Scale bars: 2 μm (upper) and 500 nm (lower). (**B**) Quantification of total reactive oxygen species (ROS); (**C**) mitochondrial ROS (mtROS); (**D**) lipid peroxidation product malondialdehyde (MDA); (**E**) total superoxide dismutase (SOD) enzymatic activity; (**F**) relative mitochondrial DNA (mtDNA) copy number; (**G**) total ATP content in BAT homogenates. (**H**–**J**) relative mRNA expression of genes related to biogenesis (Nrf1, Tfam), mitophagy (Pink1, Parkin) and genes regulating mitochondrial dynamics (Mfn1, Mfn2, Drp1); (**K**–**O**) representative Western blot and corresponding densitometric quantifications showing protein levels of NRF1, TFAM, total DRP1, and p-DRP1 Ser616 (**K**–**M**), and markers of mitophagic flux including the LC3-II/LC3-I and p62 degradation (**N**,**O**). Tubulin was used as a loading control for Western blots. Data are presented as mean ± SD with individual data points shown (*n* = 6 mice per group for physiological and molecular assays; *n* = 3 mice per group for Western blot). Statistical significance was determined by one-way ANOVA followed by Tukey’s post hoc test (* *p* < 0.05, ** *p* < 0.01, *** *p* < 0.001).

Following the activation of the SIRT1–PGC-1α axis, we evaluated whether Cpn could comprehensively restore mitochondrial quality control (MQC) in BAT. At the structural level, transmission electron microscopy (TEM) showed that Cpn treatment markedly ameliorated the severe ultrastructural damage induced by LL, including cristae disruption and vacuolation (Figure 4A). Functionally, Cpn significantly reduced the elevated levels of total reactive oxygen species (ROS) and mitochondrial reactive oxygen species (mtROS), as well as the lipid peroxidation marker malondialdehyde (MDA); restored the LL-suppressed activity of superoxide dismutase (SOD); and rescued the depleted mitochondrial DNA (mtDNA) copy number (Figure 4B–F), indicating a systemic alleviation of oxidative stress and mitochondrial biogenesis. At the molecular level, Cpn restored mitochondrial homeostasis by correcting the LL-induced dysregulation of genes governing mitochondrial dynamics (Mfn1, Mfn2, Drp1) and those involved in mitochondrial biogenesis (Tfam, Nrf1) (Figure 4H,J). This transcriptional rescue was corroborated at the protein level, where Cpn reversed LL-induced alterations in key PGC-1α-regulated proteins involved in mitochondrial biogenesis (NRF1, TFAM) and dynamics DRP1 (Figure 4K–M). Notably, while total DRP1 protein levels were significantly reduced in LL-exposed BAT and restored by Cpn, the phosphorylation of DRP1 at Ser616 relative to total DRP1 remained unchanged across all groups (Figure 4L,M), indicating that Cpn normalized the expression of the fission machinery without inducing aberrant hyperactivation of the residual protein.

Furthermore, Cpn reactivated the impaired mitophagic flux in BAT. Cpn rescued the LL-suppressed expression of key mitophagy genes (Pink1, Parkin) at the transcriptional level (Figure 4I), which was confirmed by a parallel restoration of downregulated PINK1 and PARKIN protein levels. Functionally, it promoted the degradation of the autophagy substrate p62 and increased the LC3-II/LC3-I ratio (Figure 4N,O), collectively indicating enhanced clearance of damaged mitochondria.

Notably, despite this comprehensive restoration of mitochondrial structure, redox balance, and turnover, the steady-state ATP content in BAT remained comparable across all groups (Figure 4G). This finding underscores that Cpn’s primary effect is not to elevate baseline ATP production, but to restore mitochondrial metabolic resilience—the capacity to maintain energy homeostasis and meet acute energetic demands, which aligns with the observed improvement in systemic cold tolerance.

### 2.5. SIRT1 Is Indispensable for the Cordycepin-Mediated Restoration of Mitochondrial and Metabolic Function

**Figure 5:**
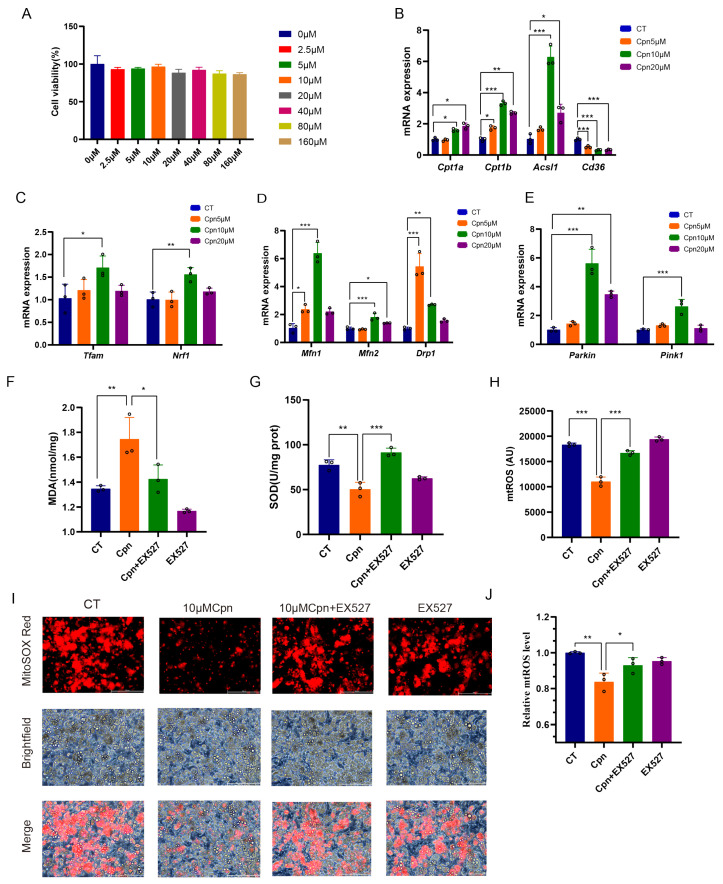
The inhibition of SIRT1 abrogates the beneficial effects of cordycepin on mitochondrial function and metabolic reprogramming in brown adipocytes. All experiments were performed on cultured primary brown adipocytes. Cells were pre-treated with cordycepin (Cpn, 10 μM) and/or the SIRT1-specific inhibitor EX-527 (10 μM) for 48 h. (**A**) Cell viability determined by CCK8 assay after treatment with increasing concentrations of Cpn; (**B**–**E**) dose–response analysis of Cpn on key target genes to determine its optimal working concentration; (**F**–**J**) quantification of cellular oxidative stress markers, including mitochondrial ROS (mtROS) (**H**–**J**) Scale bar: 200 μm, malondialdehyde (MDA) (**F**), and superoxide dismutase (SOD) activity (**G**). Data are presented as mean ± SD with individual data points shown (*n* = 3 independent experiments for in vitro studies). Statistical significance was determined by one-way ANOVA followed by Tukey’s post hoc test (* *p* < 0.05, ** *p* < 0.01, *** *p* < 0.001).

To definitively establish the necessity of SIRT1, we employed a pharmacological loss-of-function approach using the specific inhibitor EX-527 in primary brown adipocytes. The non-toxic, effective concentrations of Cpn were first determined using CCK-8 and dose–response assays (Figure 5A–E). Co-treatment with EX-527 significantly attenuated or abolished multiple beneficial effects of Cpn.

**Figure 6:**
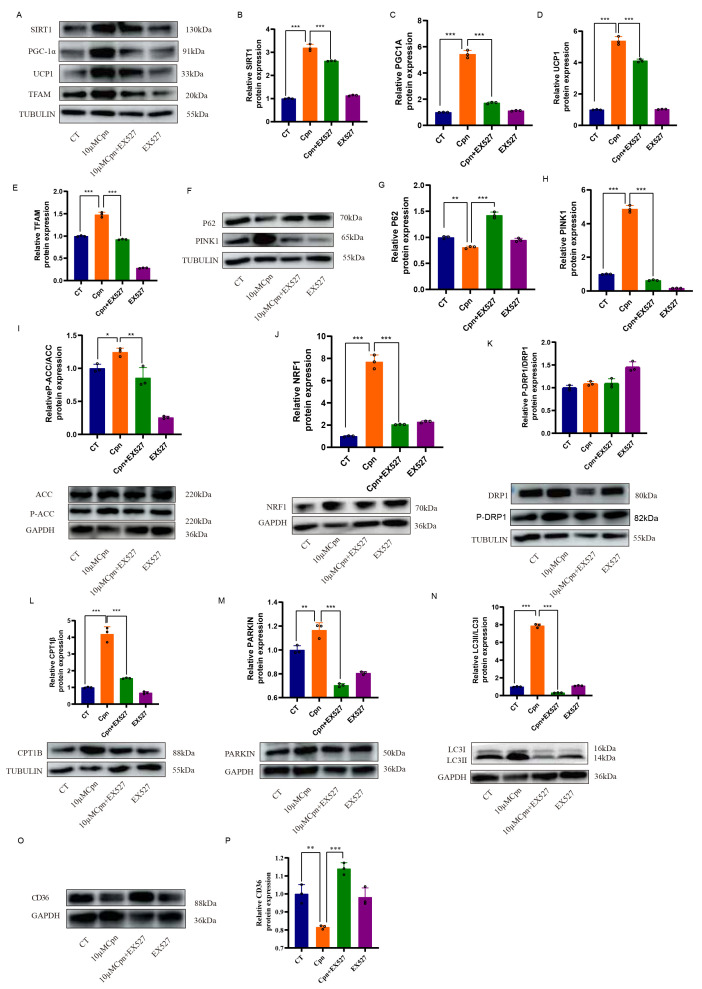
The inhibition of SIRT1 abrogates the beneficial effects of cordycepin on mitochondrial function and metabolic reprogramming in brown adipocytes. All experiments were performed on cultured primary brown adipocytes. Cells were pre-treated with cordycepin (Cpn, 10 μM) and/or the SIRT1-specific inhibitor EX-527 (10 μM) for 48 h. (**A**–**P**) The total SIRT1 protein levels and representative Western blots for key proteins involved in PGC-1α downstream signaling (p-ACC, TFAM, NRF1, CPT1β, DRP1, P-DRP1, CD36) and mitophagy (PINK1, PARKIN, LC3B, p62). Corresponding densitometric quantification of the Western blot results. Tubulin and GAPDH were used as a loading control for Western blots. Data are presented as mean ± SD with individual data points shown (*n* = 3 independent experiments for in vitro studies). Statistical significance was determined by one-way ANOVA with Tukey’s post hoc test (* *p* < 0.05, ** *p* < 0.01, *** *p* < 0.001).

Specifically, EX-527 blocked: (i) the Cpn-mediated upregulation of key PGC-1α target proteins involved in fatty acid oxidation (p-ACC, CPT1β, CD36), mitochondrial biogenesis (NRF1, TFAM), and dynamics (DRP1), while the p-DRP1 Ser616/total DRP1 ratio remained unchanged; (ii) the activation of mitophagic flux, as evidenced by the reversal of the Cpn-induced restoration of PINK1/PARKIN levels, the LC3-II/LC3-I ratio, and p62 degradation (Figure 6A–P); and (iii) the amelioration of oxidative stress markers, including reductions in mtROS and MDA and an increase in SOD activity (Figure 5F–J).

These results demonstrate that SIRT1 activity is an indispensable core mediator for the mitochondrial protective and metabolic reprogramming effects of Cpn. While EX-527 did not completely abrogate every measured parameter—suggesting potential minor contributions from SIRT1-independent pathways—this observation does not diminish the essential role of SIRT1 in the Cpn-mediated rescue.

## 3. Discussion

Mitochondrial function is central to brown adipose tissue (BAT), underpinning its adaptive thermogenesis, redox homeostasis, and metabolic flexibility [44]. To sustain this high bioenergetic demand, BAT relies on a robust mitochondrial quality control (MQC) system, which maintains homeostasis by coordinating the removal of damaged components via mitophagy with the replenishment of the mitochondrial network through biogenesis [45]. The dysregulation of this coordinated MQC is a recognized driver of lipid accumulation, thermogenic failure, and pathological BAT whitening [14].

This study demonstrates that constant light (LL) exposure, an environmental circadian disruptor, triggers pathological whitening of BAT by inducing a severe mismatch between substrate supply and oxidative capacity. Specifically, LL disrupts mitochondrial quality control (MQC), which concurrently promotes CD36-mediated fatty acid uptake and impairs CPT1-mediated oxidation. This imbalance leads to ectopic lipid accumulation, ultimately resulting in thermogenic failure and tissue whitening. A key finding of this work is that the natural product cordycepin effectively reverses this entire pathological cascade.

A key advance of this work is the identification of SIRT1 as a primary molecular target mediating cordycepin’s protection. SIRT1, an NAD^+^-dependent deacetylase, is a master regulator of cellular energy metabolism and stress adaptation, orchestrating mitochondrial biogenesis, fatty acid oxidation, and antioxidant defense largely through the deacetylation and activation of PGC-1α [46,47,48,49,50]. It was observed that LL exposure significantly reduced SIRT1 protein levels and increased PGC-1α acetylation in BAT, indicating suppression of this critical axis—effects that were effectively reversed by cordycepin.

A dual mechanism for SIRT1 activation by cordycepin is further delineated. First, molecular docking predicted stable binding of cordycepin within the SIRT1 catalytic domain, a prediction directly confirmed by surface plasmon resonance (SPR) analysis demonstrating specific binding with moderate affinity. Kinetic analysis suggested a dynamic, reversible interaction. Second, in cellular models, cordycepin significantly elevated the NAD^+^/NADH ratio, thereby increasing the availability of the essential co-substrate for SIRT1 activity. The functional necessity of SIRT1 was definitively established using the specific inhibitor EX-527, which abolished the broad spectrum of cordycepin’s downstream benefits. Together, these data establish SIRT1 as an indispensable core mediator of the therapeutic effects of cordycepin.

Activation of SIRT1 subsequently drives a comprehensive reprogramming of the mitochondrial quality control network. Mitochondrial biogenesis is essential for replenishing the mitochondrial pool and constitutes a critical component of MQC [51]. This process is primarily orchestrated by the transcriptional coactivator PGC-1α and its downstream effectors, nuclear respiratory factor 1 (NRF1) and mitochondrial transcription factor A (TFAM) [52]. It was found that LL exposure significantly downregulated PGC-1α, NRF1, and TFAM in BAT, consistent with the observed mitochondrial depletion. Cordycepin treatment effectively restored their expression, concurrently increasing levels of CPT1 β, the rate-limiting enzyme for mitochondrial fatty acid oxidation. Critically, the SIRT1-specific inhibitor EX-527 abolished the cordycepin-induced upregulation of CPT1 β, NRF1, and TFAM in primary brown adipocytes. Collectively, these results establish the SIRT1–PGC-1α axis as a core pathway through which cordycepin restores mitochondrial oxidative capacity and BAT function.

However, restoring mitochondrial biogenesis alone is insufficient to resolve persistent oxidative and lipotoxic stress [53]. A competent MQC system also requires the complementary capacity to efficiently remove damaged mitochondria. It was therefore investigated whether cordycepin restores mitophagy. The PTEN-induced kinase 1 (PINK1)–Parkin pathway serves as the principal mechanism mediating mitophagy. Upon mitochondrial depolarization, PINK1 accumulates on the outer mitochondrial membrane [54,55], where it recruits cytosolic Parkin. Parkin, in turn, ubiquitinates outer membrane proteins [56], marking the mitochondria for autophagic clearance via adaptors such as p62 [57,58]. The results of this study demonstrate that cordycepin reactivates this LL-suppressed mitophagic program in BAT, as evidenced by restored PINK1 and Parkin expression, an elevated LC3-II/LC3-I ratio, and enhanced clearance of p62. This restoration was dependent on SIRT1 activity, as co-treatment with EX-527 significantly attenuated these cordycepin-induced effects. These findings suggest cordycepin does not non-specifically stimulate general autophagy but rather preferentially rescues stalled mitophagy, a key action for restoring overall mitochondrial fitness.

AMP-activated protein kinase (AMPK) is a master sensor of cellular energy status and shares extensive crosstalk with SIRT1. AMPK directly phosphorylates ACC at Ser79, leading to its inhibition and subsequent reduction in malonyl-CoA levels, thereby relieving CPT1-mediated fatty acid oxidation [38,40]. Consistent with this, our data show that cordycepin restored AMPK phosphorylation (p-AMPK Thr172) in LL-exposed BAT, which likely contributed to the observed normalization of ACC phosphorylation, malonyl-CoA levels, and CPT1β expression. Importantly, AMPK and SIRT1 form a positive feedback loop: AMPK activation increases NAD^+^ levels via upregulation of NAMPT, enhancing SIRT1 activity [20,28]. The concurrent activation of both kinases by cordycepin therefore establishes a coordinated AMPK–SIRT1 signaling axis that integrates energy sensing with mitochondrial quality control to restore metabolic homeostasis in BAT. Given the established crosstalk between AMPK and the mechanistic target of rapamycin (mTOR) in regulating cellular metabolism, the potential involvement of the AMPK–mTOR axis in cordycepin’s effects warrants consideration. AMPK activation typically suppresses mTOR complex 1 (mTORC1) signaling under conditions of energy stress, which in turn influences mitochondrial biogenesis, lipid metabolism, and adipocyte differentiation [59,60]. While mTOR activity was not directly assessed in the present study, it remains plausible that AMPK-mediated suppression of mTOR signaling could contribute to the metabolic benefits of cordycepin in BAT. Future studies using specific inhibitors or genetic approaches will be necessary to delineate the role of the AMPK–mTOR axis in this context.

Our SPR analysis showed that cordycepin binds to SIRT1 with moderate affinity and fast dissociation kinetics (Figure 3I,J), a kinetic profile often associated with allosteric modulators [61]. To explore the structural basis of this interaction, we performed molecular docking simulations using the crystal structure of human SIRT1 (PDB ID: 5BTR). The simulation predicted that cordycepin binds to SIRT1, forming conventional hydrogen bonds with key residues including ASN 226, ARG 446, GLU 230, and PRO 447. Notably, the predicted binding residues include GLU 230 and ASN 226, which are located within or near the well-characterized N-terminal STAC-binding domain (SBD), where Glu230 has been shown to be critical for allosteric activation by resveratrol and synthetic STACs [62,63]. Given these findings, we speculate that cordycepin may interact with SIRT1 in a manner that involves the N-terminal region, potentially inducing conformational changes that enhance its deacetylase activity. Nevertheless, the precise binding mode of cordycepin on SIRT1 requires further experimental validation.

An intriguing observation was that despite extensive mitochondrial damage, steady-state ATP levels in BAT remained unchanged. This suggests that LL does not abolish basal energy production but rather depletes metabolic resilience—the capacity to acutely upregulate oxidative phosphorylation in response to energetic challenges such as cold stress. By restoring the MQC network, Cordycepin enhances not only mitochondrial quantity and baseline function but, crucially, this functional reserve and adaptive capacity.

**Figure 7:**
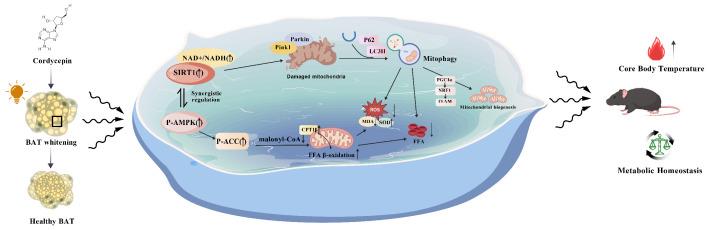
Schematic summary of the molecular mechanism underlying the ameliorative effect of cordycepin on constant light-induced metabolic dysfunction (created with BioGDP.com).

This study delineates a pathogenic axis linking light exposure to metabolic dysfunction: constant light–SIRT1 suppression–collapse of mitochondrial quality control–loss of metabolic resilience (Figure 7). Importantly, this study demonstrates that cordycepin directly interacts with SIRT1 and enhances its activity, thereby restoring mitochondrial homeostasis and counteracting metabolic injury. While our data support a functional interaction between cordycepin and SIRT1, definitive proof of direct catalytic activation would require additional biochemical and genetic studies, such as SIRT1 knockdown or rescue experiments with catalytically inactive mutants. Nevertheless, these findings provide a novel mechanistic basis for potential intervention in light pollution-related metabolic disorders.

Several limitations of the present work should be acknowledged and point to important future directions. First, the precise mechanism by which cordycepin elevates SIRT1 protein levels—whether through enhanced transcription/translation or improved protein stability—remains to be elucidated.Second, while our pharmacological inhibition with EX-527 demonstrates SIRT1 dependence, the indispensable role of SIRT1 warrants further validation using tissue-specific knockout models or genetic approaches such as SIRT1 knockdown in primary brown adipocytes with rescue experiments using catalytically inactive mutants. Third, a direct pharmacokinetic correlation between the in vitro concentration (10 μM) and the in vivo dose (50 mg/kg) used in this study was not established, as plasma or tissue concentrations of cordycepin were not measured. This limits our ability to directly link the effective concentration observed in cultured cells to the dose administered in animals. Future studies incorporating detailed pharmacokinetic profiling—including measurements of cordycepin absorption, distribution, and tissue bioavailability—would help to better define the relationship between in vitro efficacy and in vivo dosing, and to support the translational development of cordycepin as a therapeutic agent for metabolic disorders. Future studies should aim to deepen these mechanistic insights, explore the broader applicability of SIRT1-targeting natural products to other metabolic disorders, and advance their translational development.

## 4. Materials and Methods

### 4.1. Animals

Eight-week-old male C57BL/6J mice (body weight 21–23 g) were obtained from Beijing Vital River Laboratory Animal Technology Co., Ltd. (Beijing, China). Mice were housed under specific pathogen-free (SPF) conditions in a controlled environment with a 12 h light/dark cycle (lights on from 8:00 am to 8:00 pm), a temperature of 24 ± 2 °C, and a relative humidity of 40–60%. Standard chow and water were provided ad libitum. All experimental procedures were reviewed and approved by the Institutional Animal Care and Use Committee (IACUC) of the Academy of Military Medical Sciences (Approval No. AMMS-04-2025-009, Tianjin, China).

Following a one-week acclimation period, mice were randomly assigned to three experimental groups (n = 11 per group): LD group (Control)—maintained under a standard 12 h:12 h light–dark cycle; LL group (Constant Light)—exposed to continuous light (24 h light); and LL+Cpn group (Cordycepin Intervention)—exposed to continuous light (24 h light) and administered cordycepin orally. Mice in the LL+Cpn group received a daily oral gavage of cordycepin (Cpn, 50 mg/kg body weight; purity ≥ 98%, Shanghai Yuanye Bio-Technology Co., Ltd., Shanghai, China) dissolved in sterile distilled water. Mice in the LD and LL groups received an equal volume of vehicle (sterile distilled water) daily. The light intensity in all housing cages was maintained at approximately 400 Lux. The intervention continued for five consecutive weeks. The constant light intensity of 400 Lux was selected based on preliminary studies from our laboratory demonstrating that this intensity effectively induces BAT whitening and metabolic dysfunction in mice. The dose of cordycepin (50 mg/kg) for in vivo experiments was selected based on preliminary dose-finding studies conducted in our laboratory. The 50 mg/kg dose showed superior efficacy in ameliorating constant light-induced BAT whitening, consistent with our previously published findings [36]. Therefore, 50 mg/kg was chosen as the optimal dose for mechanistic investigation in the present study.

At the end of the treatment period, mice were fasted for 6 h. Blood samples were then collected via retro-orbital puncture under brief isoflurane anesthesia. Subsequently, mice were euthanized by cervical dislocation. Interscapular brown adipose tissue (BAT) was rapidly dissected, weighed, and processed. One portion of the tissue was snap-frozen in liquid nitrogen and stored at −80 °C for subsequent molecular analyses. Another portion was fixed in 4% paraformaldehyde (in phosphate-buffered saline, PBS) for histological examination.

### 4.2. Acute Cold Tolerance Test

Thermogenic capacity was assessed via an acute cold tolerance test. Mice were fasted for 5 h prior to the experiment. Animals from each group were then individually placed into a pre-cooled chamber maintained at −5 °C without bedding, food, or water. To minimize isolation stress during the test, mice were acclimated in pairs but tested individually.

Core body temperature was measured at designated time points (0, 30, 60, 90, 120, 150, 180, 210, 240, 270 min) using a rectal probe connected to a digital thermometer (Physitemp BAT-12). Simultaneously, the skin temperature over the interscapular brown adipose tissue (BAT) depot was monitored using an infrared thermal camera (FLIR E6). Survival time in the cold environment was recorded for each mouse.

### 4.3. Body Composition Analysis

Prior to the terminal procedures (euthanasia), mice were fasted for 6 h. Body composition was analyzed in vivo using a dedicated small-animal dual-energy X-ray absorptiometry (DEXA) scanner (InAlyzer, Medikors, Seongnam, Republic of Korea). Total fat mass and lean body mass were determined from whole-body scans using the manufacturer’s proprietary software.

### 4.4. Histological Analysis

#### 4.4.1. Hematoxylin and Eosin (HE) Staining

Brown adipose tissue (BAT) samples fixed in 4% paraformaldehyde were dehydrated through a graded ethanol series, cleared in xylene, and embedded in paraffin. Serial sections (4 μm thick) were cut using a microtome and mounted on glass slides. After deparaffinization in xylene and rehydration through a descending ethanol series, sections were stained with hematoxylin and eosin (HE) following standard protocols. Finally, sections were dehydrated, cleared, and cover-slipped with a neutral mounting medium. Images were acquired using a light microscope (Leica DM500, Leica Microsystems, Wetzlar, Germany).

#### 4.4.2. Oil Red O (ORO) Staining

Fresh BAT samples were embedded in optimal cutting temperature (OCT) compound, snap-frozen, and stored at −80 °C. Frozen sections (10 μm thick) were prepared using a cryostat, air-dried, and fixed in 4% paraformaldehyde for 20 min. Sections were then rinsed with 60% isopropanol and stained with a freshly filtered Oil Red O working solution for 15 min at room temperature. Excess stain was removed by washing in 60% isopropanol. Nuclei were counterstained with hematoxylin. Sections were mounted with an aqueous mounting medium (glycerol-gelatin) and imaged under a light microscope. The area positive for Oil Red O staining was quantified using ImageJ software (National Institutes of Health, USA, Version 1.53q).

### 4.5. Transmission Electron Microscopy (TEM)

Brown adipose tissue (BAT) was dissected and immediately cut into 1 mm^3^ pieces. Tissue pieces were fixed overnight at 4 °C in 2.5% glutaraldehyde (prepared in 0.1 M phosphate buffer, pH 7.4). After rinsing with the same buffer, samples were post-fixed with 1% osmium tetroxide for 2 h at room temperature. Samples were then dehydrated through a graded series of ethanol (50%, 70%, 90%, and 100%), followed by infiltration and embedding in epoxy resin (Eponate 12 (Ted Pella, Redding, CA, USA)). Ultrathin sections (60–80 nm) were cut using an ultramicrotome, collected on copper grids, and double-stained with uranyl acetate and lead citrate. Mitochondrial ultrastructure was observed and imaged using a transmission electron microscope (Hitachi HT7800, Tokyo, Japan).

### 4.6. Biochemical Marker Assays

Biochemical parameters in serum and tissue homogenates were measured using commercial assay kits according to the manufacturers’ protocols. Serum lipid profiles, including total cholesterol (TC), triglycerides (TG), high-density lipoprotein cholesterol (HDL-C), and low-density lipoprotein cholesterol (LDL-C), were analyzed using kits from Nanjing Jiancheng Bioengineering Institute. Levels of free fatty acids (FFA) and malondialdehyde (MDA), as well as the activity of superoxide dismutase (SOD), were determined using kits from Biyuntian Biotechnology Co., Ltd. (Shanghai, China). SIRT1 deacetylase activity was assessed with a fluorometric assay kit (ab156065; Abcam, Cambridge, UK). The intracellular NAD^+^/NADH ratio was measured using a corresponding detection kit from Beyotime Biotechnology.

### 4.7. Determination of Malonyl-CoA Levels in Mouse Brown Adipose Tissue

Malonyl-CoA levels in mouse brown adipose tissue (BAT) were determined using a double-antibody one-step sandwich ELISA kit (Jiangsu Meimian Industrial Co., Ltd., Taizhou, China). Briefly, BAT samples were homogenized in pre-chilled phosphate-buffered saline (PBS) containing protease inhibitors at a ratio of 1:9 (*w*/*v*). The homogenate was centrifuged at 4 °C and 5000× *g* for 10 min, and the supernatant was collected. Following the manufacturer’s instructions, standard solutions or test samples (diluted 5-fold) were added to the appropriate wells, followed by HRP-conjugated detection antibody, and the plate was incubated at 37 °C for 60 min. The plate was then washed five times, and substrate solution was added and incubated in the dark for 15 min. After the reaction was stopped, the optical density (OD) was measured at a wavelength of 450 nm. Sample concentrations were calculated based on the standard curve, converted to actual concentrations according to the dilution factor, and expressed in ng/mg of tissue weight.

### 4.8. ATP Content Measurement

Total ATP levels in BAT tissue were measured using an ATP Assay Kit (Beyotime Biotechnology Shanghai, China) according to the manufacturer’s instructions. Briefly, tissue was homogenized in ice-cold lysis buffer and centrifuged at 12,000× *g* for 5 min at 4 °C. The resulting supernatant was collected, and ATP concentrations were determined using a standard curve (0.01–10 μmM ATP) with a luminometer (Synergy H1, BioTek Instruments, Winooski, VT, USA). Protein concentration in the same supernatant was measured using a BCA assay. ATP levels were normalized to protein content and expressed as nmol/mg protein.

### 4.9. Primary Brown Adipocyte Culture and Treatment

Primary brown adipocyte precursor cells were isolated from the interscapular brown adipose tissue (BAT) of 5–7-day-old neonatal C57BL/6J mice. Briefly, minced tissue was digested at 37 °C for 30–45 min in DMEM/F12 medium containing 1.5 mg/mL collagenase type II (McLean Biochemical Technology, Shanghai, China) with gentle agitation. The digest was sequentially filtered through 100 μm and 70 μm cell strainers. After centrifugation, the cell pellet was resuspended in growth medium (DMEM/F12 supplemented with 10% fetal bovine serum [FBS; Gibco] and 1% penicillin–streptomycin) and seeded into culture plates. Cells were maintained under standard conditions in a cell culture incubator at 37 °C with 5% CO_2_.

Upon reaching 80–90% confluence, differentiation was induced by replacing the growth medium with differentiation medium containing 0.5 mM IBMX, 1 μM indomethacin, 1 μM dexamethasone, 2 μg/mL insulin, and 1 nM triiodothyronine (T3) in DMEM/F12 with 10% FBS. After 48 h, this medium was replaced with maintenance medium (DMEM/F12 with 10% FBS, 2 μg/mL insulin, 1 nM T3, and 1 μM rosiglitazone), which was refreshed every two days. Cells were considered fully differentiated after 7–9 days, as indicated by the accumulation of multilocular lipid droplets.

The working concentration of cordycepin (10 μM) for in vitro experiments was selected by comparing three concentrations (5, 10, and 20 μM) in primary brown adipocytes. As shown in Figure 5B–E, while no clear dose-dependent effect was observed, 10 μM consistently produced the most robust and statistically significant effects on the mRNA expression of key genes involved in mitochondrial function and metabolism. This concentration was also confirmed to be non-cytotoxic by CCK-8 assay (Figure 5A–E). Therefore, 10 μM was selected as the optimal concentration for subsequent mechanistic studies. For experimental treatments, fully differentiated brown adipocytes were incubated with the indicated concentrations of cordycepin (Cpn) and/or the SIRT1-specific inhibitor EX-527 (DC7127; Shanghai Dongcang Biotechnology, Shanghai, China) for specified durations prior to harvest and analysis.

### 4.10. Cell Viability Assay

The effect of cordycepin (Cpn) on cell viability was assessed using a Cell Counting Kit-8 (CCK-8; Tianjin Yijun Biotechnology, Tianjin, China). Fully differentiated primary brown adipocytes were seeded in 96-well plates at a density of 5 × 10^3^ cells per well and allowed to adhere overnight. Cells were then treated with culture medium containing various concentrations of Cpn for 48 h. After treatment, 10 μL of CCK-8 reagent was directly added to each well containing 100 μL of the original culture medium. The plates were incubated at 37 °C for 1 h. The absorbance of each well at 450 nm was measured using a microplate reader (BioTek uQuant, Winooski, VT, USA). Cell viability was expressed as a percentage relative to the untreated control group.

### 4.11. Reactive Oxygen Species (ROS) Detection

#### 4.11.1. Detection of Total and Mitochondrial ROS in BAT Tissue

Intracellular total ROS levels in brown adipose tissue (BAT) were measured using the fluorescent probe 2’,7’-dichlorodihydrofluorescein diacetate (DCFH-DA; Box Biotech, Redwood City, CA, USA). Mitochondrial superoxide levels in BAT were detected using MitoSOX™ Red mitochondrial superoxide indicator (Solarbio, Beijing, China). Briefly, tissue homogenates were incubated with the respective probes according to the manufacturer’s protocols. Fluorescence was measured using a microplate reader (BioTek Synergy H1) with excitation/emission settings of 488/525 nm for DCFH-DA and 510/580 nm for MitoSOX™ Red.

#### 4.11.2. Detection of Mitochondrial ROS in Cultured Cells

Mitochondrial superoxide in primary brown adipocytes was assessed using MitoSOX™ Red (Solarbio). Following the indicated treatments, cells were incubated with 5 μM MitoSOX™ Red in serum-free medium at 37 °C for 30 min in the dark. Cells were then washed twice with warm phosphate-buffered saline (PBS). Fluorescence images were acquired using a fluorescence microscope (DFC450 C, Leica Microsystems, Wetzlar, Germany) with appropriate filters.

### 4.12. Mitochondrial DNA Copy Number Analysis

Total DNA was extracted from BAT tissue using the TIANamp Genomic DNA Kit (TIANGEN, DP304), following the manufacturer’s instructions. Quantitative real-time PCR (qPCR) was performed to determine the relative mitochondrial DNA (mtDNA) copy number, using the mitochondrial gene COX-II as a mitochondrial marker and the single-copy nuclear gene β-globin as a reference. The relative mtDNA copy number was calculated using the $2^{-\Delta \Delta C_{t}}$ method, with the LD group serving as the control. Primer sequences are listed in Table A1.

### 4.13. RNA Extraction and Quantitative Real-Time PCR (qRT-PCR)

Total RNA was extracted from brown adipose tissue (BAT) or cultured primary brown adipocytes using TRIzol reagent (Sangon Biotech, Shanghai, China), following the manufacturer’s instructions. RNA concentration and purity were determined by measuring the absorbance at 260 nm and 280 nm using a microvolume spectrophotometer (NanoDrop™ One, Thermo Fisher Scientific, Waltham, MA, USA). Only samples with an A260/A280 ratio between 1.8 and 2.0 were used for subsequent analysis. For each sample, 1 μg of total RNA was reverse-transcribed into cDNA using the PrimeScript RT Reagent Kit (Takara Bio, R045A, Kusatsu, Japan).

Quantitative PCR (qPCR) was performed using a QuantStudio™ 5 Real-Time PCR System (Applied Biosystems, Foster City, CA, USA) with SYBR Green chemistry. Each 20 μL reaction mixture contained 10 μL of 2× SYBR Green Master Mix, 1 μL of cDNA template, and 0.5 μM of each gene-specific forward and reverse primer. The thermal cycling protocol consisted of an initial denaturation at 95 °C for 30 s, followed by 40 cycles of denaturation at 95 °C for 5 s and annealing/extension at 60 °C for 30 s. A melting curve analysis was performed at the end of each run to verify primer specificity. The 36B4 (Arbp) gene served as the endogenous reference for normalization. Relative gene expression levels were calculated using the comparative $2^{-\Delta \Delta C_{t}}$ method. All primer sequences used are listed in Appendix A.

### 4.14. Western Blot Analysis

Proteins were extracted from brown adipose tissue (BAT) or cultured cells using RIPA lysis buffer (Solarbio) supplemented with protease and phosphatase inhibitor cocktails. Protein concentrations were determined using a bicinchoninic acid (BCA) assay kit (Solarbio) according to the manufacturer’s protocol.

Equal amounts of protein (20–40 μg per lane) were separated by SDS-PAGE on 8–12% gels and then electrophoretically transferred onto polyvinylidene difluoride (PVDF) membranes (Merck Millipore, Burlington, MA, USA). After transfer, the membranes were blocked with 5% (*w*/*v*) non-fat milk in Tris-buffered saline with 0.1% Tween-20 (TBST) for 2 h at room temperature. The membranes were then incubated overnight at 4 °C with specific primary antibodies diluted in the blocking buffer. Primary antibody details are listed in Appendix B.

Following three 10 min washes with TBST, the membranes were incubated with horseradish peroxidase (HRP)-conjugated secondary antibodies for 1 h at room temperature. After another series of TBST washes, protein bands were visualized using an enhanced chemiluminescence (ECL) substrate kit (Millipore) and imaged with a chemiluminescence imaging system (Thermo Fisher Scientific iBright FL1500). Band intensities were quantified using ImageJ software (National Institutes of Health, USA, Version 1.53q) and normalized to the corresponding loading control (TUBULIN or GAPDH).

### 4.15. Molecular Docking

Molecular docking was performed to investigate the potential binding of cordycepin to the target protein. The three-dimensional structure of human SIRT1 (PDB ID: 5BTR) was obtained from the Protein Data Bank (PDB: https://www.rcsb.org/ accessed on 19 September 2025) [64]. Water molecules and non-essential heteroatoms were removed from the protein structure prior to docking.

The structure of cordycepin was prepared using OpenBabel software (version 3.1.1) [65]. Both the protein and ligand structures were subsequently processed with AutoDockTools-1.5.6. This included adding hydrogen atoms, assigning charges, and defining the rotatable bonds of the ligand. The docking search space (grid box) was centered on the putative active site of the protein. Docking simulations were then conducted using AutoDock Vina 1.2.7 [66] with default parameters. The binding affinity was evaluated based on the calculated docking score (kcal/mol).

The resulting docking poses were visualized and analyzed using PyMOL (version 2.3.4) and Discovery Studio Visualizer 2021.

### 4.16. Surface Plasmon Resonance (SPR) Analysis

The direct interaction between cordycepin (Cpn) and recombinant human SIRT1 protein was analyzed by surface plasmon resonance (SPR) using a Biacore T200 system (Cytiva, Marlborough, MA, USA). Recombinant human SIRT1 (MedChemExpress, HY-P71596, Monmouth Junction, NJ, USA) was immobilized on a CM5 sensor chip via amine coupling using the standard EDC/NHS chemistry. A series of CPN solutions at varying concentrations (0.625–10 μM, prepared in running buffer) were injected over the sensor surface at a constant flow rate of 30 μm L/min. Binding and dissociation phases were monitored in real time. Sensorgram data were globally fitted to a 1:1 Langmuir binding model using the Biacore Insight Evaluation Software (v6.0, Cytiva) to derive the kinetic parameters: association rate constant ($k_{a}$), dissociation rate constant ($k_{d}$), and equilibrium dissociation constant ($K_{D}$).

### 4.17. Statistical Analysis

Data are expressed as mean ± standard deviation (SD). Statistical analyses were conducted using GraphPad Prism software (version 8.0). Statistical significance was determined by one-way ANOVA followed by Tukey’s post hoc test. Survival curves were compared using the log-rank (Mantel–Cox) test. A *p* value of less than 0.05 was considered statistically significant.

## Data Availability

The original contributions presented in this study are included in the article. Further inquiries can be directed to the corresponding author.

## Abbreviations

The following abbreviations are used in this manuscript:

ALAN	Artificial Light at Night
LL	Constant Light
LD	Light/Dark
BMI	Body Mass Index
BAT	Brown Adipose Tissue
SIRT1	Sirtuin 1
PGC-1α	Peroxisome proliferator-activated receptor gamma coactivator 1-alpha
NAD^+^	Nicotinamide Adenine Dinucleotide
NADH	Nicotinamide Adenine Dinucleotide
CPT1β	Carnitine Palmitoyltransferase 1 Beta
ACC	Acetyl-CoA Carboxylase
p-ACC	Phosphorylated ACC
ROS	Reactive Oxygen Species
mtROS	Mitochondrial Reactive Oxygen Species
MDA	Malondialdehyde
SOD	Superoxide Dismutase
ATP	Adenosine Triphosphate
Cd36	Cluster of Differentiation 36
Fasn	Fatty Acid Synthase
Atgl	Adipose Triglyceride Lipase
Hsl	Hormone-Sensitive Lipase
Mfn1/2	Mitofusin 1/2
Drp1	Dynamin-Related Protein 1
Tfam	Mitochondrial Transcription Factor A
Nrf1	Nuclear Respiratory Factor 1
pink1	PTEN-Induced Kinase 1
LC3B	Microtubule-associated protein 1A/1B-light chain 3
SPR	Surface Plasmon Resonance
TEM	Transmission Electron Microscopy
MQC	Mitochondrial Quality Control
mtDNA	Mitochondrial DNA
Cpn	Cordycepin
EX-527	the selective SIRT1 inhibitor EX-527
P-DRP1	Phosphorylated Dynamin-Related Protein 1
AMPK	5’-Adenosine Monophosphate-Activated Protein Kinase
P-AMPK	Phosphorylated 5’-Adenosine Monophosphate-Activated Protein Kinase

## Appendix A

**Table A1:** Sequences of primers for qPCR.

Gene	Species	Sequence ($5^{\prime }$–$3^{\prime }$)
Fasn	Mouse	F:GGAGGTGGTGATAGCCGGTAT R:TGGGTAATCCATAGAGCCCAG
Atgl	Mouse	F:GCCACTCACATCTACGGAGC R:CCACGGATGGTCTTCACCAG
Prdm16	Mouse	F:CCACCAGCGAGGACTTCAC R:GGAGGACTCTGTAGCTCGAA
Hsl	Mouse	F:TATGGAGTGAGGGTGCCAGA R:ATGGTCCTCTGCCTCTGTCC
Acaca	Mouse	F:AGGCGGATATCTGCTGAGAC R:GGAGTGCTGGTTTAGCTCCA
Cpt1α	Mouse	F:GTCTGGCTCTACCATGACGG R:CCCTGTTCCGATTCGTCCAA
Cpt1β	Mouse	F:GCACACCAGGCAGTAGCTTT R:CAGGAGTTGATTCCAGACAGGTA
Acsl1	Mouse	F:AGGACTCGGCATGTGACAAA R:ACACCGCAGCAGAATCAGAA
Cd36	Mouse	F:TGGAGGCATTCTCATGCCAG R:TTGCTGCTGTTCTTTGCCAC
Tfam	Mouse	F:GAGCAGCTAACTCCAAGTCAG R:GAGCCGAATCATCCTTTGCCT
Nrf1	Mouse	F:GCTGTCCGATATCCTGGTGG R:GGTGGGGGACAGATAGTCCT
Mfn1	Mouse	F:GATAAAGTCCTCCCCAGCGG R:GCATGGGCCAGCTGATTAAC
Mfn2	Mouse	F:GCGCCAGTTTGTGGAATACG R:CGGGTGATGTCAACTTGCTG
Drp1	Mouse	F:GCCTCAGATCGTCGTAGTGG R:TCAACTCCATTTTCTTCTCCTGT
Parkin	Mouse	F:AGAGCACCTGGAAACAGGAG R:CTCCACACCTCCTCCTCTTC
Pink1	Mouse	F:TGGCTGAAGAGCAACAAGGT R:AAGTCCAGGGCGTCCTAAAT
36b4	Mouse	F:CTGCACTCTCGCTTTCTGGA R:ACGCGCTTGTACCCATTGAT
COX-II	Mouse	F:GCCGACTAAATCAAGCAACA R:CAATGGGCATAAAGCTATGG
β-globin	Mouse	F:GAAGCGATTCTAGGGAGCAG R:GGAGCAGCGATTCTGAGTAG

## Appendix B

**Table A2:** Antibodies used in the Western blotting analysis in the study.

Antibody	Cat No.	Manufacturer
SIRT1	BS45100	bioworlde
PGC1-α	BS40401	bioworlde
P-ACC	BS4210P	bioworlde
ACC	BS90018	bioworlde
UCP1	EPR20381	abcam
PARKIN	AF0235	affinity
PINK1	EPR20730	abcam
LC3B	EPR18709	abcam
P62	EPR4844	abcam
TFAM	AF0531	affinity
NRF1	AF5298	affinity
DRP1	DF7037	affinity
TUBULIN	BS45073	bioworlde
GAPDH	MB66349	bioworlde
CD36	ab252922	abcam
P-DRP1	ab314755	abcam
P-AMPK	2535T	CST
AMPK	66536-1-Ig	proteintech
